# Supplementary material for: An interactive mass spectrometry atlas of histone posttranslational modifications in T-cell acute leukemia
Source: Sci Data. 2022 Oct 15;9:626. doi: 10.1038/s41597-022-01736-1 (PMC9569336; doi:10.1038/s41597-022-01736-1)
Supplement: Supplementary file 1 — Supplementary File 2 [file 41597_2022_1736_MOESM1_ESM.pdf]

Materials and methods

Western blot. Histones from 6 x 10^6 cells were extracted via the DA extraction method. After extraction, the histone concentration was measured via the BCA protein assay kit. An equal number of histones for each cell line was further prepped for western blot. 5x Laemmli buffer with β-mercaptoethanol was added to the samples in a 1:4 ratio and incubated for 10 minutes at 95°C for denaturation. To perform gel electrophoresis, 20 µl of the samples was loaded on two 9-16% Mini-PROTEAN TGX Precast Protein Gels (Bio-Rad: 456-1103) and 4 µl of the Page Ruler Plus Prestained protein ladder (Perbio: 26620). To identify H3K27me3, the anti-H3K27me3 antibody (07-449; Millipore) was added to one blot (1/1000 in 5% milk/TBST) and incubated overnight. To normalize against the H3 level, the H3 antibody (ab1791; Abcam) was added to the other blot (1/1000 in 5% milk/TBST). After addition of anti-rabbit antibody, both blots were visualized on the Amersham Imager 680 (GE healthcare) via SuperSignal West Dura Extended Duration Substrate.

Flow cytometry. A 0,3 million cell pellet of each cell line was resuspended in 100 µl wash buffer (PBS with 2% BSA) before the cells were stained using the Live/Dead Dye efluor 506 (Invitrogen: 65-0866-14). After 30 minutes incubation in the dark at 4°C, the cells were washed with PBS and centrifuged for 5 minutes at 1500rpm at 4°C and the supernatant was drained. Next, the cells were fixated and permeabilized (Invitrogen eBioscience FOXP3/Transcription) to open the membranes for intracellular staining by incubating the cells for 30 minutes at room temperature with 0.5 ml Fix/Perm. After washing the pellets twice with Perm/milliQ water (1/10), the cells were resuspended in 300µl wash buffer and split into three tubes. For each cell line, one tube was stained with H3K27me3 Ab (Millipore; 07-449), another with the H3 Ab (Abcam; ab1791), and a third with only the secondary Ab as negative control to detect background fluorescence and to know the threshold. After 30 minutes incubation in the dark at 4°C, pellets were washed twice before the cells were resuspended in 100µl PBS. Next, the secondary antibody (Thermofisher; AF488) was added and the cells were incubated in the dark at 4°C for 30 minutes. Pellets were washed twice before the cells were resuspended in 150µl PBS and the signal was measured on the BD LSR II (Biosciences). The data was analyzed using FlowJo. Four gating selections were made: SSC vs FSC to deselect debris, SSC-H vs SSC-A to select singlets, AmCyan vs FSC to select living cells, histogram of FITC to visualize the H3 and H3K27me3 level. The measured level of H3K27me3 was normalized against the measured level of H3.

Results

Flow cytometry normalization calculations.

	**H3K27me3**	**H3**	**Normalized H3K27me3 = H3K27me3/H3**
HSB-2	10429	7595	1,37314
PEER	14288	8882	1,608647
JURKAT	15356	10791	1,423038
MOLT-16	9119	5124	1,779664
TALL-1	15414	15372	1,002732
RPMI-8402	17741	16516	1,074171
LOUCY	6621	9333	0,709418
HPB-ALL	9276	11920	0,778188
