# Supplementary material for: An interactive mass spectrometry atlas of histone posttranslational modifications in T-cell acute leukemia
Source: Sci Data. 2022 Oct 15;9:626. doi: 10.1038/s41597-022-01736-1 (PMC9569336; doi:10.1038/s41597-022-01736-1)
Supplement: Supplementary file 2 — Supplementary File 1 [file 41597_2022_1736_MOESM2_ESM.pdf]

Supplementary File 1

Randomization scheme.

Due to the high number of samples, samples were randomized into multiple batches for histone extraction (letter A to C) and propionylation (pink or purple).
When the number of histones extracted from a sample was too low,
an extra histone extraction was performed in either batch D or E.

Histone extraction: batch A → E
Propionylation steps: batch 1 or batch 2

	REPLICATE					
**CELL LINES**	1	2	3	4	5	6
ALL-SIL	A > E	B > E	C	A	B	C
CCRF-CEM	C	A > E	B	C	A > D	B
CUTTL-1	A	B > D	C	A	B	C
DND-41	B > D	C > E	A > D	B	C	A > D
HPB-ALL	C	A	B	C	A	B
HSB-2	A	B	C	A	B	C
JURKAT	A	B	C	A	B	C
KARPAS-45	C	A	B	C	A	B
KARPAS-45 JC	A	B > E	C	A	B	C
KE-37	A	B	C	A	B > D	C
KOPTK-1	B > E	C	A > E	B	C	A > D
LOUCY	C	A	B > E	C	A	B
MOLT-4	A	B	C	A	B	C
MOLT-16	B	C	A	B	C	A
P12-ICHIKAWA	C	A > E	B	D	A	B
PEER	C	A	B	C	A	B
PER-117	C	A > D	B > D	C	A	B
PF-382	B > E	C > D	A > D	B	D	A > D
RPMI-8402	B	C	A	B	C	A
SUPT-11	B	C	A	B	C	A
TALL-1	B	C	A	B	C	A
